# Recombinant Pure PDGF Improves Aesthetic Results and Patient Satisfaction Following RF Microneedling: A Prospective, Randomized, Controlled Clinical Trial

**DOI:** 10.1111/jocd.70425

**Published:** 2025-09-12

**Authors:** Samuel E. Lynch, Shawn T. Huxel, Rhonda Bond, Julie Biron, Michael Gold

**Affiliations:** ^1^ Lynch Regenerative Medicine Franklin Tennessee USA; ^2^ TN Clinical Research Center Nashville, TN USA

**Keywords:** aesthetics, dermatology, PDGF, platelet‐derived growth factor, pure PDGF, radiofrequency microneedling

## Abstract

**Background:**

Platelet‐Derived Growth Factor BB (PDGF) is a potent stimulator of tissue regeneration. It has received FDA approvals in four medical therapeutic indications for tissue regeneration. Recently, the use of pure PDGF has also been introduced for the promotion of skin rejuvenation and regeneration in aesthetic indications.

**Aims:**

Evaluate the efficacy and safety of the topical application of pure PDGF compared to standard of care following radiofrequency (RF) microneedling.

**Methods:**

In this evaluator‐blinded, prospective, randomized, controlled clinical trial, subjects between 30 and 60 years of age received a single treatment of RF microneedling, followed by application of either standard of care (Aquaphor) or pure PDGF. Participants were evaluated at 7 and 30 days post‐procedure by a board‐certified dermatologist (MG) to determine the Clinical Global Aesthetic Improvement Score (CGAIS), and by Canfield Image Analysis (IA) software to assess seven aesthetic parameters. Patient‐reported outcome measures (PROMs) were also obtained at 7 and 30 days following treatment.

**Results:**

Pure PDGF treatment resulted in greater improvement in the CGAIS as determined at 7 and 30 days compared to the standard of care group. Additionally, PDGF treatment outperformed the standard of care group in 6 of 7 outcomes in the objective image analysis. PROMs indicated that participants treated with pure PDGF had an overall better experience than participants receiving standard of care. No serious adverse effects were reported.

**Conclusions:**

This prospective, randomized, controlled clinical trial shows the application of pure PDGF possesses an excellent safety profile and improves facial skin quality 30 days post RF microneedling more effectively than the current standard of care.

## 1 Introduction

Platelet‐derived growth factor (PDGF) plays an important role in wound healing, tissue regeneration, and rejuvenation [1, 2, 3]. During healing, PDGFs are released from alpha granules of activated platelets and synthesized by local cells at the wound site. PDGF binds to cell surface receptors (PDGFRa/b) present on cells of mesenchymal origin to activate cell signaling cascades driving key physiological processes underlying tissue regeneration such as cellular mitogenesis, differentiation, chemotaxis, cell survival, and angiogenesis [4, 5, 6]. The presence of these cell surface receptors is upregulated following injury [7], further accentuating the effects of PDGF. Receptors for PDGF are found on dermal fibroblasts, mesenchymal stem cells (MSCs), smooth muscle cells, pericytes, glial cells, mesangial cells, osteoblasts, chondroblasts, and other cells of mesenchymal origin, allowing for spatially restricted cell‐specific responses [8, 9, 10]. Due to PDGF's clinical efficacy and safety [11, 12, 13, 14, 15, 16], it has gained FDA approval when used for multiple tissue regeneration indications throughout the body, including promoting the healing of skin wounds, regeneration of oral and maxillofacial tissues including maxillary and mandibular bone, gingival and mucosa, and bone regeneration to achieve arthrodesis in the ankle and hindfoot [17, 18].

Radiofrequency (RF) microneedling is a procedure which combines the benefits of microneedling and radiofrequency energy together into a single unified form of dermal treatment. During the treatment, a device with tiny needles is used to create micro‐injuries in the skin, while simultaneously delivering radiofrequency energy [19, 20]. This energy penetrates deep into the dermis, stimulating collagen and elastin production, resulting in tighter, smoother, and more youthful‐looking skin [19, 21]. The fractional RF microneedling technology of Morpheus8 is a versatile treatment modality capable of skin resurfacing, tightening, and subdermal adipose remodeling with a strong safety profile in all skin types, making it widely appealing to both clinicians and patients [22].

While both PDGF and RF microneedling have each shown clinical efficacy at improving skin quality, the safety and efficacy of combining these two modalities for improving regenerative skin aesthetics remain unknown. Therefore, we performed an evaluator‐blinded, prospective, randomized, controlled human clinical trial to characterize the efficacy and safety of the novel post‐procedural application of a recombinant pure PDGF serum (rhPDGF‐BB; herein referred to as PDGF) following Radiofrequency (RF) Microneedling with the Morpheus8 platform in female subjects between 30 and 60 years old.

## 2 Materials and Methods

### 2.1 Patient Selection

Twelve subjects aged between 35 and 65 years with signs of skin aging that met the inclusion criteria were selected for the study (Table S1). Patients were randomized into standard of care (*n* = 4 participants) or experimental group (*n* = 8 participants) following a 2:1 treatment to placebo design appropriate for gathering greater information on the treatment group [23].

### 2.2 Trial Design

This clinical trial was a prospective, single‐center, two‐arm, evaluator‐blinded pilot trial with a 30‐day posttreatment follow‐up period. Eligible study subjects were identified during a screening visit prior to or on the same day as the Baseline (Day 0) and were randomized into one of the two study groups. Prior to treatment, the treatment area was evaluated to determine the baseline characteristics and received a topical anesthetic (20% Benzocaine, 10% Lidocaine, and 10% Tetracaine). At the baseline visit, participants underwent a single RF microneedling treatment with the Morpheus8 fractional microneedling device (InMode, Irvine, CA, USA) according to the manufacturer's instructions for use. Participants recorded their experiences during the 12 h following treatment in a diary. The efficacy of the treatment was evaluated in all participants at follow‐up visits 1, 3, 7, and 30 days post‐procedure (Table S2).

### 2.3 Materials

The test treatment consisted of 300 ug/mL of recombinant pure PDGF‐BB in a sterile physiologic solution (Lynch Regenerative Medicine, Franklin, Tennessee, USA). The standard of care consisted of an emollient (Aquaphor; CT, USA).

### 2.4 PDGF Or Aquaphor Application

The study subjects who were randomized to the test group had PDGF applied immediately following the RF microneedling. The PDGF (up to 1.0 mL) was delivered onto the skin surface through a sterile blunt needle (0.5–1″ long; 16–20 Ga) and spread over the treatment area with a sterile gloved finger. Ten minutes following the application of the PDGF, emollient was then applied to the treated area. Subjects randomized to the standard of care group received the emollient post‐microneedling.

### 2.5 Objective Image Analysis

Face photographs were obtained using VISIA‐CR (Canfield Scientific, USA) pre‐microneedling procedure, post‐microneedling procedure, and 1 to 30 days post‐microneedling procedure. Three images were captured (frontal, right side 45°, and left side 45°) at each time point. Makeup and jewelry were removed prior to photographing subjects, and headbands were used to keep hair away from the face. Seven parameters were evaluated, including pore count, pore fractional area, texture fractional area, spot count, wrinkle coarse area, wrinkle count, and wrinkle mean thickness. An Area of Interest (AOI) mask template was applied to all applicable images. For the Frontal view template, vertical guidelines were placed at the outer canthus, and a horizontal guideline was placed at the right inner canthus to delineate the edges of the AOI. For the Left Oblique and Right Oblique view image templates, the full cheek area and undereye were masked. Horizontal guidelines were placed at the outer canthus, and vertical guidelines were placed at the inner canthus. A vertical guideline was placed at the inner eyebrow. 2‐D Image Analysis algorithms detected wrinkles, visible spots, pore detection, and texture.

### 2.6 Blinded Global Aesthetic Improvement Evaluation

The efficacy of PDGF compared to standard of care was evaluated by a blinded evaluator (MG) reviewing standardized photographs 7 to 30 days post‐procedure according to the Clinical Global Aesthetic Improvement Scale (CGAIS), using a 5‐point scale ranging from very much improved (1) to worse (5) (Table S3).

### 2.7 Patient‐Reported Outcomes

Participants were asked to report the general level of treatment discomfort on a scale of 0 (none) to 10 (maximum intolerable pain) using the universal pain assessment tool. Participants completed the initial pain evaluation in the office within 10 min posttreatment and application of either standard of care or PDGF. Additional pain evaluation was collected via subject diary, dispensed at Day 0 and collected at Day 1. Self‐assessed tolerability and pain were recorded by participants at three time points: 1 to 2 h posttreatment, 6 to 8 h posttreatment, and 12 to 16 h posttreatment. Other patient‐reported outcome measures were recorded throughout the study related to subjective improvement in skin quality.

### 2.8 Statistical Analysis

Differences in CGAIS between standard of care and PDGF at 7 and 30 days posttreatment was determined with an unpaired two‐tailed t‐test (GraphPad Prism). Statistical significance with respect to the PDGF compared to standard of care is declared if the two‐sided *p* < 0.05. Data are presented as mean ± standard deviation.

### 2.9 Ethical Approval of Human Subjects

The trial was approved by relevant local ethics committees and Institutional Review Board (IRB) (IRB#00000971). The trial was conducted in compliance with the Declaration of Helsinki and International Council for Harmonisation of Technical Requirements for Pharmaceuticals for Human Use (ICH) guidelines. All participants provided written informed consent prior to the initiation of the study.

## 3 Results

### 3.1 Study Participants

A total of 13 female subjects were recruited for the study. One subject was lost to follow‐up, resulting in a final study population of 12 participants. The age of enrolled subjects was between 43 and 64 years (mean 53.6 years). During the baseline visit, the Fitzpatrick skin type of each subject was recorded. Two of the participants (17%) were assessed as Fitzpatrick Type 1; 6 (50%) as Type 2; 3 (25%) as Type 3; and one (8%) as Type 4. The Glogau classification was also recorded, with six (50%) of the participants classified with “moderate” skin damage and six (50%) of the participants classified as “advanced”. None of the participants were classified with “mild” or “severe” Glogau ratings.

### 3.2 PDGF Application Improves Global Aesthetic Improvement Scores

**FIGURE 1:**
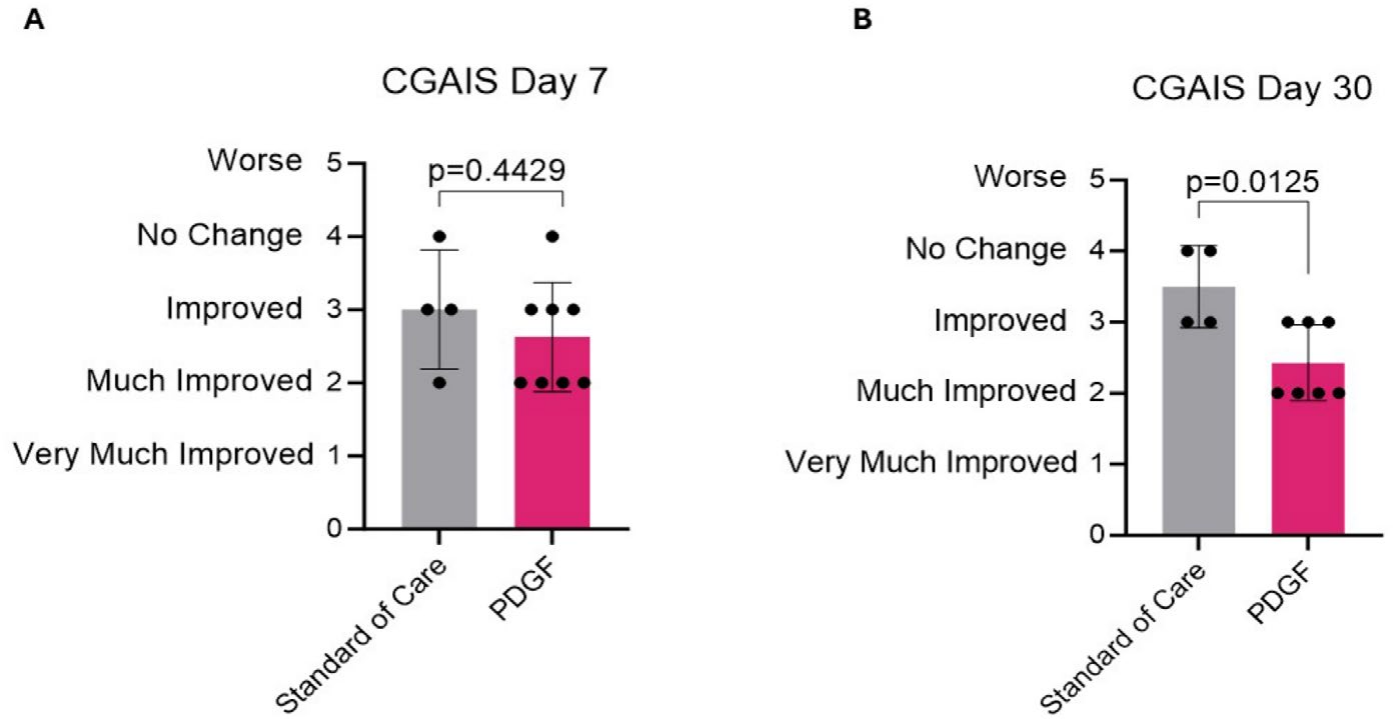
Pure PDGF significantly improves CGAIS 30 days following RF microneedling. CGAIS 7 days (A) and 30 days (B) following RF microneedling treatment. Data are represented as mean ± SD. *N* = 4 for standard of care; *N* = 8 for PDGF.

**FIGURE 2:**
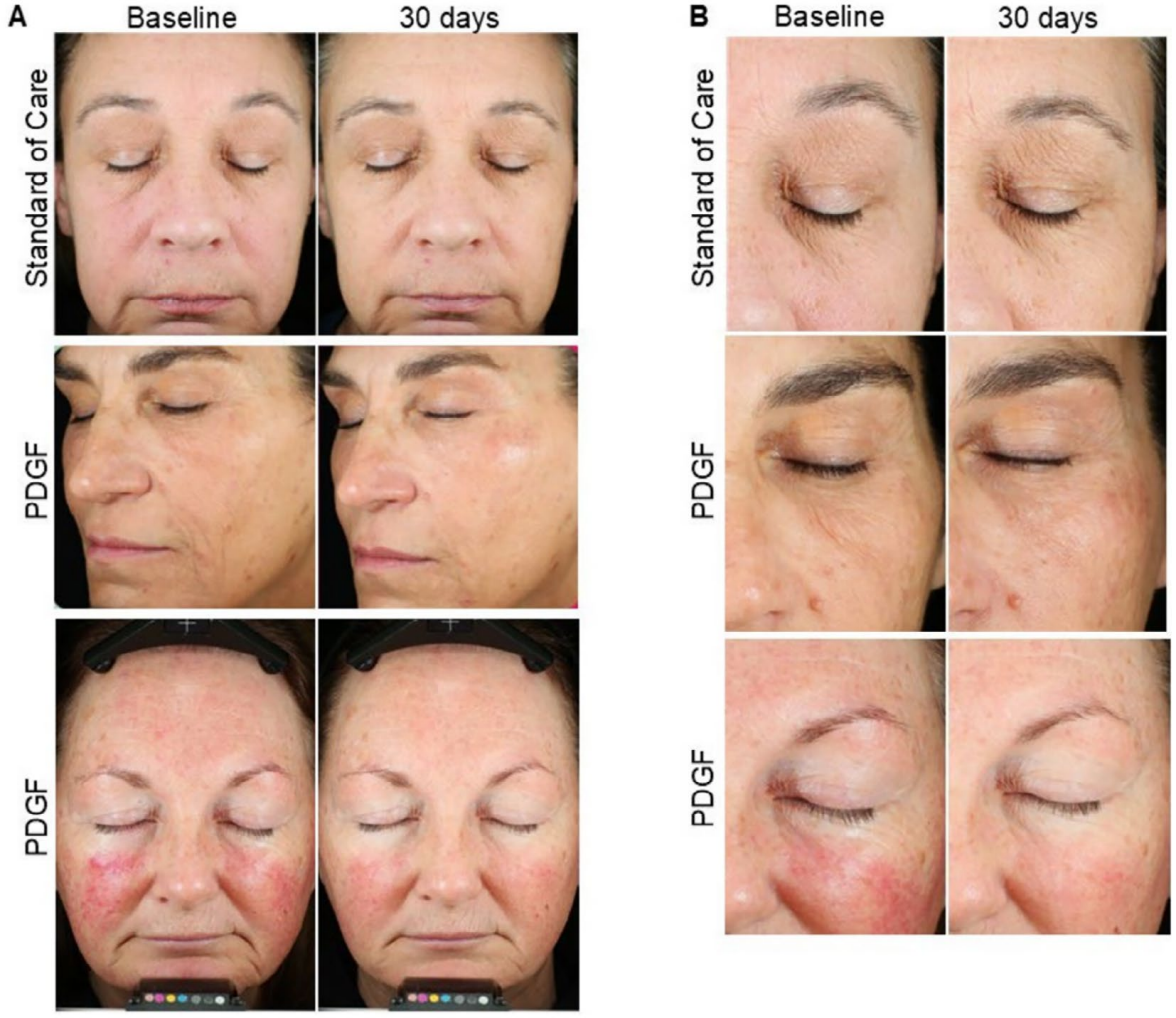
Pure PDGF improves facial healing and aesthetic outcomes following RF microneedling treatment. (A) Representative images of participants undergoing RF microneedling treatment immediately followed by standard of care treatment (top row) or PDGF treatment (middle and bottom rows) at baseline and 30 days post‐RF microneedling. (B) Closer views of photomicrographs from participants depicted in panel (A).

To determine the clinical efficacy of PDGF on improving recovery from Morpheus8 microneedling, we performed a blinded evaluation of photographs using the CGAIS at baseline and on visits five (day 7) and six (day 30). Participants who received the standard of care (positive control) had a mean CGAIS of 3.0 at 7 days post‐Morpheus8 treatment, which increased at day 30, indicating a worsening of skin appearance during this time period (Figure 1). Participants receiving PDGF post‐Morpheus8 procedure, on the other hand, had a lower CGAIS at day 7 (2.6 vs. 3.0 in controls), which further improved at day 30 to 2.4 (Figure 1). Comparing the CGAIS between standard of care and PDGF indicated no significant difference on day 7 (Figure 1A); however, significant differences between the standard of care group and participants receiving PDGF were observed 30 days post‐procedure. Specifically, participants receiving PDGF scored a full point better out of the 5‐point scale compared to participants receiving the standard of care (Figure 1B). Representative photographs showed a reduction in redness, fewer fine lines and wrinkles, and better skin quality overall in PDGF‐treated individuals compared to participants receiving the standard of care, which typically showed minimal changes from baseline to day 30 (Figure 2). These data indicate that receiving PDGF immediately following RF microneedling treatment further improves facial skin appearance over the 30‐day study period.

### 3.3 PDGF Improves Skin Appearance as Measured Using Image Analysis

**FIGURE 3:**
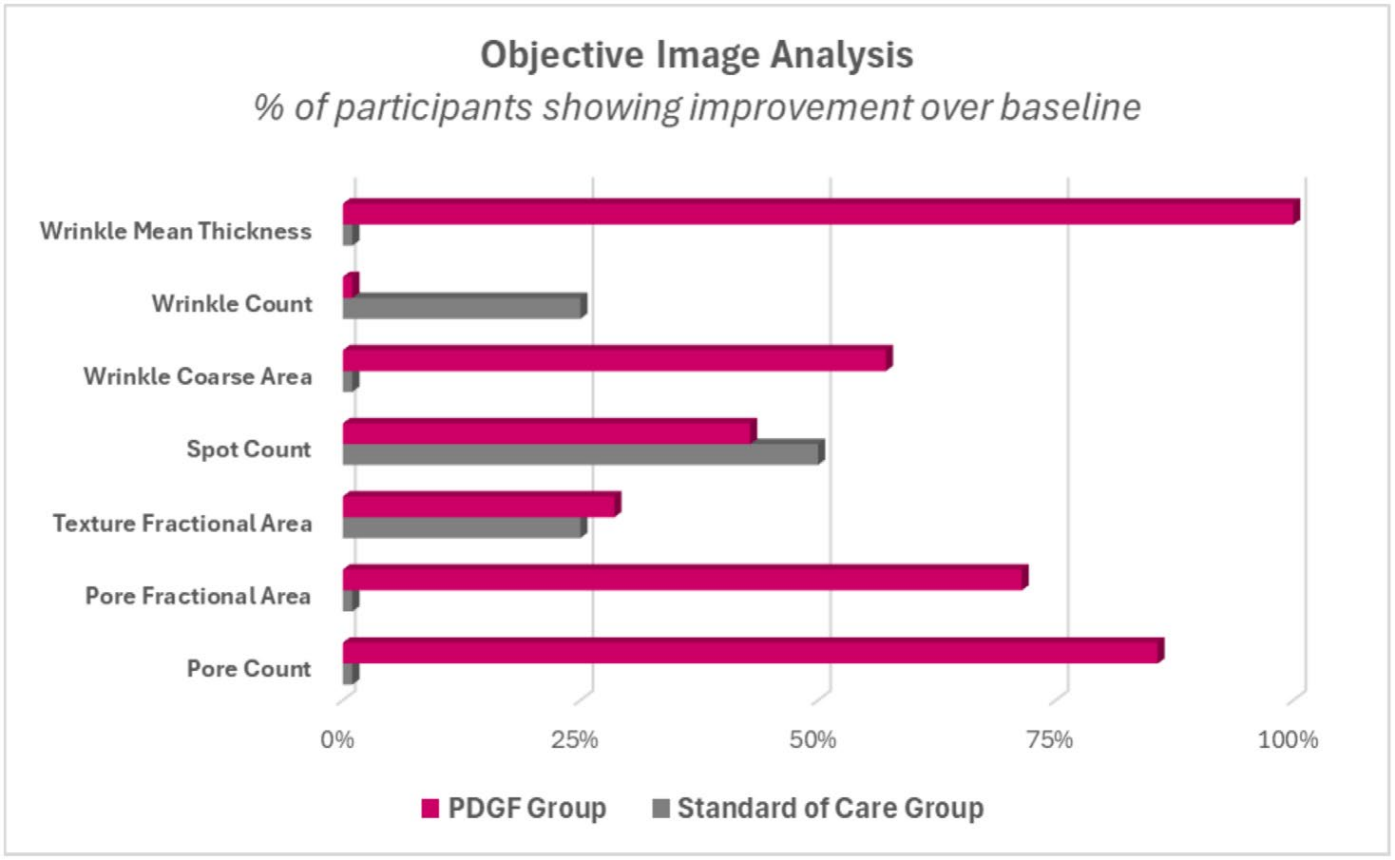
Pure PDGF improves aesthetic outcomes 30 days post‐RF microneedling. Objective image analysis comparing photomicrographs of participants treated with previous standard of care or PDGF from baseline to 30 days post‐procedure. Data are represented as % of participants showing improvement over baseline. *N* = 4 participants for standard of care; *N* = 7 participants for PDGF.

**FIGURE 4:**
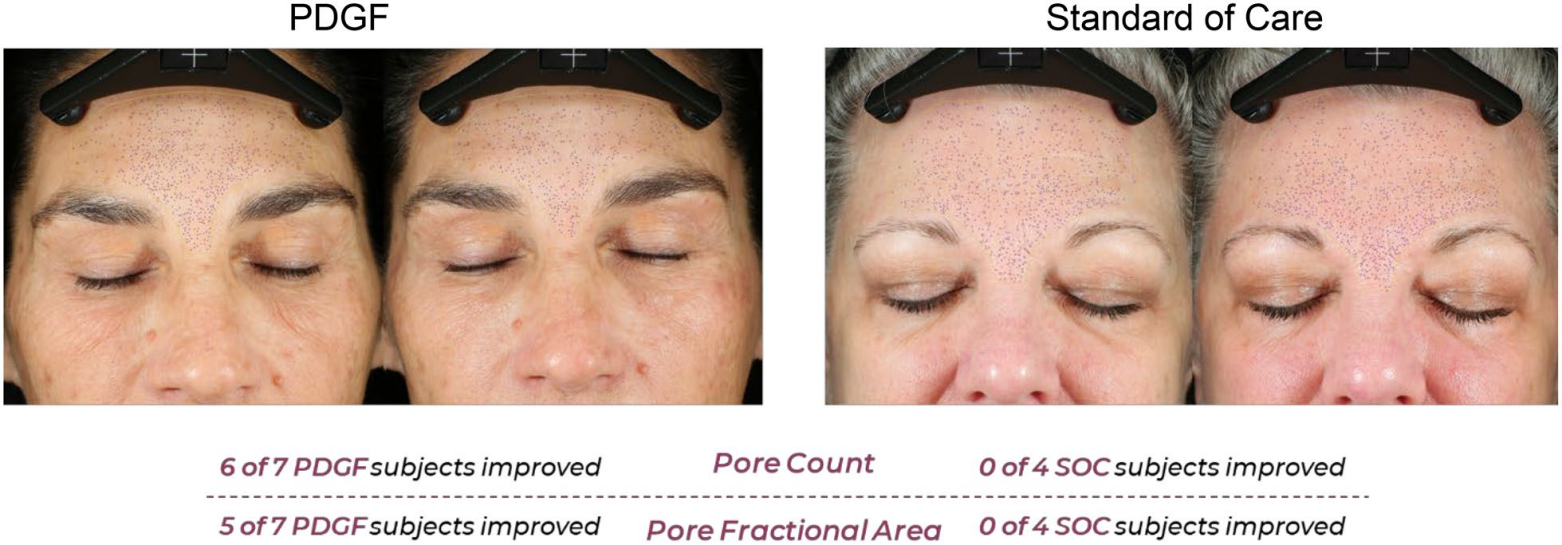
PDGF improves pore count and pore fractional area 30‐days post‐microneedling. Representative images of participants 30 days following microneedling and treatment with either PDGF (left) or standard of care (right). Patient responsiveness is detailed below images. Purple dots represent pores used for automated counting and measuring pore fractional area.

**FIGURE 5:**
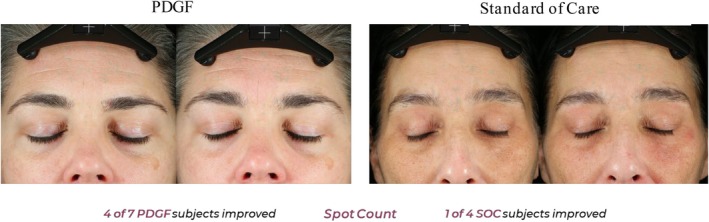
PDGF treatment improves spot count following microneedling treatment. Representative images of participants 30 days following microneedling and treatment with either PDGF (left) or standard of care (right). Patient responsiveness is detailed below images.

**FIGURE 6:**
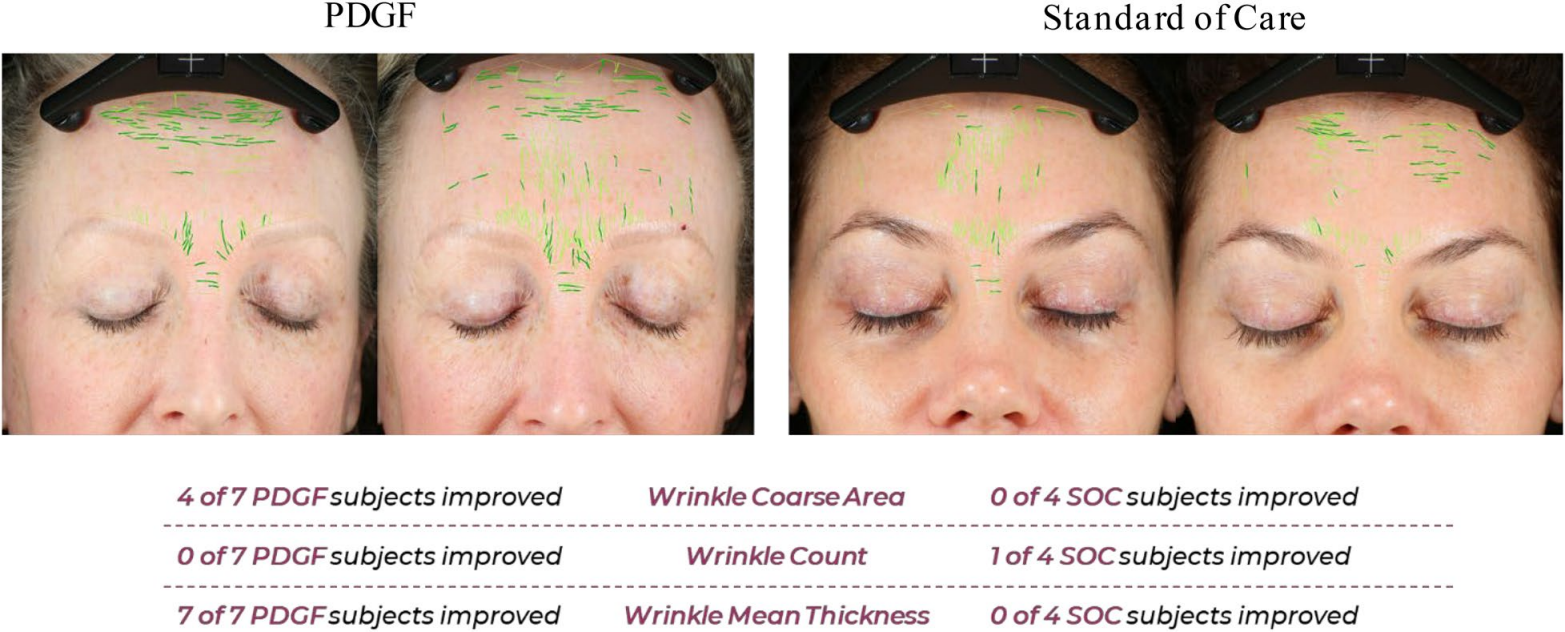
PDGF improves wrinkle coarse area and wrinkle mean thickness but not wrinkle count following microneedling. Representative images of participants 30 days following microneedling and treatment with either PDGF (left) or standard of care (right). Patient responsiveness is detailed below images. Green markings indicate wrinkles used for automated wrinkle counting and measuring wrinkle mean thickness and wrinkle coarse area.

Canfield Image Analysis was performed on study photographs at baseline and 30 days post‐procedure. Comparing frontal baseline images to 30‐day post‐procedure, improvements in the standard of care group were only observed for texture fractional area, spot count, and wrinkle count (Figure 3). Additionally, these improvements were mostly restricted to only one out of the four individuals, except for spot count, in which two participants improved over baseline. Participants receiving PDGF, in contrast, showed improvement in six out of the seven categories assessed. Moreover, a substantially higher proportion of participants from this group improved over baseline (Figure 3). When compared to the standard of care, PDGF performed better at improving all categories evaluated, except for wrinkle count (Figures 3, 4, 5, 6). Taken together, these image analysis data revealed that PDGF leads to marked improvement in objective skin appearance following RF microneedling treatment when compared to the standard of care.

### 3.4 PDGF Improves Patient's Perception of Skin Quality and Satisfaction

**FIGURE 7:**
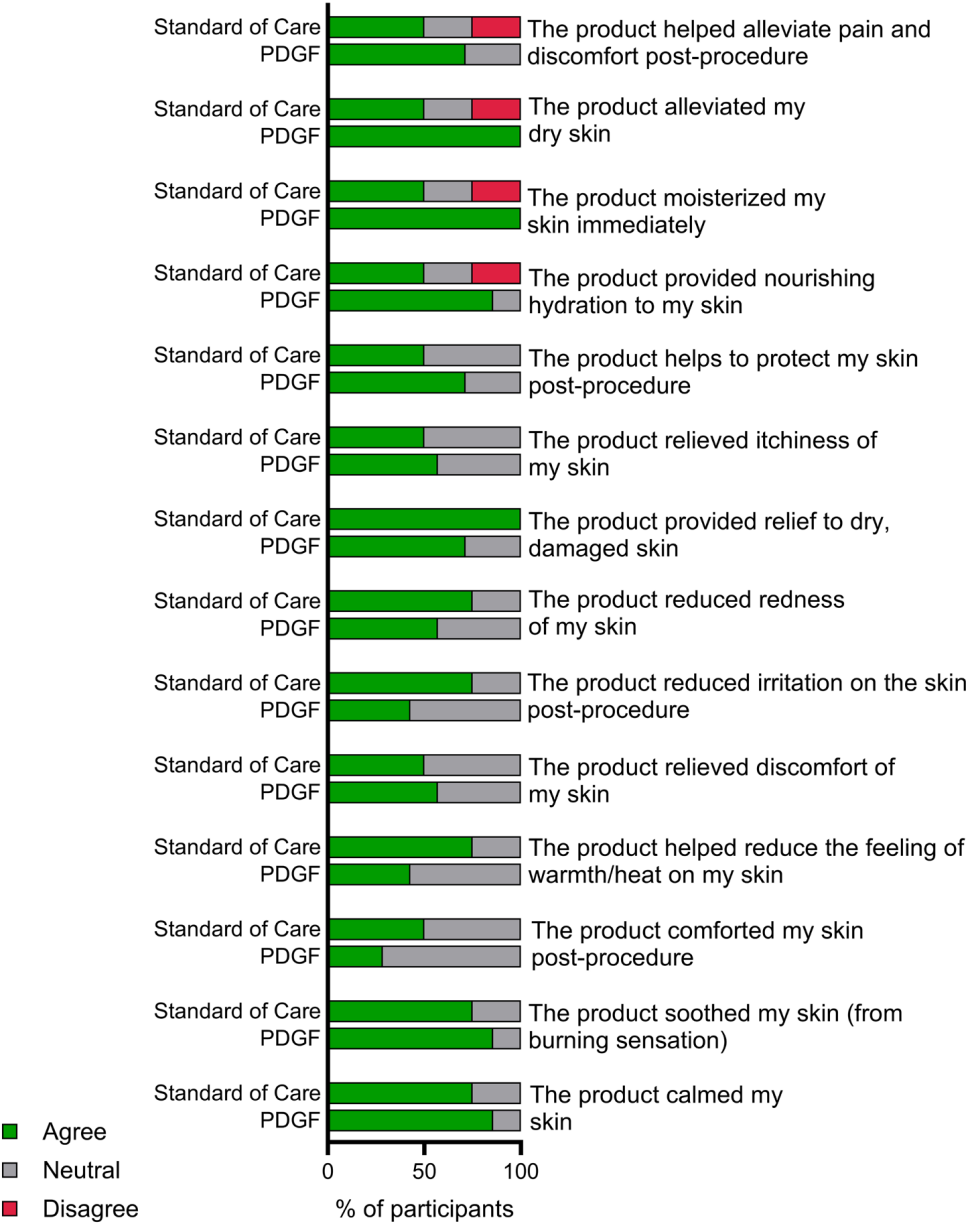
Participant satisfaction posttreatment. Participant satisfaction survey results immediately following RF microneedling treatment and application of PDGF or standard of care demonstrated that patients treated with PDGF more consistently agreed that the treatment alleviates pain and discomfort, reduces dry skin, moisturizes my skin immediately, and provides nourishing hydration compared to patients treated with the previous standard of care. Data are represented as percent of participants responding to “agree,” “neutral,” or “disagree.”

Participant's perception of their improved skin quality and posttreatment satisfaction aligned with the CGAIS and image analysis data. Regarding posttreatment satisfaction, participants were evaluated on their subjective posttreatment sensations such as skin calming effect, skin soothing effect, and alleviating post‐procedure pain. In general, participants receiving PDGF agreed that the product provided post‐procedure relief of pain, reduced irritation and redness, and was soothing to the skin (Figure 7). Conversely, participants who received the standard of care did not agree that the product soothed/cooled/calmed their skin post‐procedure (Figure 7). We further evaluated participants' perception of skin quality improvement from baseline to end of study. Patient satisfaction scores at 30 days revealed that all patients were interested in returning for a second procedure, regardless of whether they received the standard of care or PDGF. Importantly, 100% of participants in the PDGF treatment group strongly agreed that they would recommend the product to a friend or family member, while only 50% of participants receiving standard of care agreed with this statement.

### 3.5 PDGF Did Not Elicit any Adverse Events in the Study

PDGF was well tolerated, with no adverse reactions reported by the subjects, providers, or the investigators. All subjective tolerability sensations resolved after day 1 and were unremarkable for the duration of the study. All subjects indicated zero to very little pain posttreatment which, if any, resolved by Visit 3 (Day 1).

## 4 Discussion

The present study investigated the safety and efficacy of applying a recombinant pure Platelet Derived Growth Factor (PDGF) following Radiofrequency (RF) microneedling to the full face. Using objective CGAIS by a blinded board‐certified dermatologist and image analysis, coupled with participant surveys, we demonstrated that the application of PDGF following RF microneedling treatment is well tolerated and improves skin quality and healing 30 days post‐procedure with no adverse effects.

The role of platelet‐derived factors has been examined extensively in the field of aesthetics [24]. While multiple studies have demonstrated the efficacy of PRP or PRF therapy in the aesthetic field, major drawbacks are associated with its use. The variability of PRP and PRF is widely known, likely in large part due to the variability and nearly always unknown quality of the starting material (e.g., platelet counts) [25, 26, 27]. Additionally, even if platelet counts could somehow be standardized, the amount of growth factors naturally present in the body declines with age and in the presence of certain diseases such as diabetes [28, 29, 30, 31]. Therefore, the patients who could benefit from a boost in healing, such as older patients, diabetics, smokers, and others, often are the poorest candidates for use of PRP/PRF due to lower levels of growth factors present in their blood [28].

Multiple adverse events also have been reported with the use of PRP therapy, including infections, allergic reaction, inflammatory responses, serum sickness disease, and blindness [32, 33, 34, 35, 36, 37, 38, 39]. Given the variability of PRP/PRF and these adverse events, it is prudent to identify the component of PRP/PRF that is primarily responsible for the clinical benefits while avoiding the negative side effects. PDGF is such a growth factor. Recombinant pure PDGF has received four (4) FDA approvals. Of particular relevance to the field of facial skin and bone regeneration and rejuvenation, large pivotal (phase III) clinical trials in patients with poorly healing skin wounds or maxillofacial defects have shown that PDGF treatment led to significant improvements in skin healing and bone and soft tissue regeneration compared to previous standard of care [40, 41, 42, 43, 44].

From a mechanistic perspective, PDGF mediates the wound healing process through coordination and stimulation of cell survival, proliferation, and recruitment to sites of injury, as well as promoting the secretion of extracellular matrix components such as collagen, hyaluronic acid, fibronectin, and others [45, 46]. Additionally, PDGF promotes angiogenesis leading to new blood vessel formation at injured sites [1, 4]. Our findings extend these clinical indications of PDGF into the field of aesthetic medicine. Results from the present blinded prospective randomized controlled trial indicate that PDGF reduces redness and irritation and improves aesthetic results over the 30‐day study period, compared to the standard of care.

Microneedling, including RF microneedling, is known for its benefit in treating the signs of aging [47, 48, 49]. Fractional RF microneedling offers a versatile treatment modality for a wide range of dermatologic concerns and is safe for use in patients of all skin types [50]. Minimal posttreatment recovery time (compared to other energy‐based products such as laser resurfacing) and enduring results make fractional RF microneedling an increasingly attractive option for patients desiring minimally invasive options. While microneedling is appreciated as relatively safe, skin irritation remains a common complaint post‐procedure [51]. Our results provide evidence that our novel pure PDGF therapy is safe and effective at reducing post‐procedure irritation and redness associated with RF microneedling treatment compared to the currently accepted standard of care (emollient, e.g., Aquaphor). Additionally, these results translated to positive patient‐reported outcomes related to post‐procedure pain, discomfort, and healing.

There are several strengths to our current study. First, our use of randomization and blinding of a highly qualified evaluator reduces the possibility of recruitment and investigator biases. Additionally, our randomized controlled trial design allowed us to directly compare the efficacy of PDGF to the current post‐Morpheus8 standard of care. The use of objective image analysis algorithms is another strength of our study, allowing for the unbiased detection of quantitative differences between treatment groups. Finally, we incorporated patient‐reported outcomes using surveys. This allowed us to gain insight into the patient's perspective on the performance of PDGF. Limitations of our study include the use of a single site and a relatively small sample size. While our sample size was limited, this study is the first prospective randomized controlled trial evaluating the effects of pure PDGF in conjunction with microneedling of the skin and provides valuable safety data showing excellent tolerability of PDGF treatment following this procedure. Moreover, the results of this trial support the benefits of pure PDGF on skin healing and lay the foundation for additional characterization of the clinical efficacy of PDGF. Our study was also restricted to a single treatment and a 30‐day follow‐up. Future studies designed for longer follow‐ups and multiple treatments would further elucidate the long‐term results of this combination therapy [48].

In conclusion, this randomized, evaluator‐blinded, prospective controlled clinical trial demonstrates the excellent safety profile of pure PDGF, with no adverse effects reported. The trial also demonstrated the clinical benefit of the use of pure PDGF compared to previous standards of care following RF microneedling, with PDGF treatment resulting in greater reduction of irritation and redness, greater improvement in aesthetic results 30 days after treatment as assessed by both a blinded board certified evaluator and objective image analysis, and greater patient satisfaction. Taken together, these findings highlight the clinical benefits of pure PDGF in aesthetic medicine.

## Author Contributions

Dr. Michael Gold and TN Clinical Research Center had full access to all the data in the study and take responsibility for the integrity of the data and the accuracy of the data analysis, concept and design, acquisition, analysis, and interpretation of data, drafting of the manuscript, critical revision of the manuscript, statistical analysis, administrative/technical support, and supervision.

## Ethics Statement

This study complied with the principles of the requirements defined in Health Canada regulations, ICH GCP guidelines, FDA regulations at 21 CFR part 56, and HHS regulations at 45 CFR part 46. Advarra IRB carries out its functions in accordance with FDA regulations at 21 CFR parts 50, 56, 312, and 812; HHS regulations at 45 CFR part 46, subparts A‐E; good clinical practices; Health Canada regulations; and the Tri‐Council Policy Statement: Ethical Conduct for Research Involving Humans, as appropriate to the research. Advarra IRB is registered with OHRP and FDA under IRB#00000971. All participants provided written informed consent.

## Consent

Participants provided consent for their photographs to be displayed for educational or scientific purposes.

## Conflicts of Interest

Samuel E. Lynch and Shawn T. Huxel are both employees of Lynch Regenerative Medicine (LRM). Dr. Michael Gold is a consultant to LRM and received funding for this clinical study. Dr. Michael Gold, Rhonda Bond, and Julie Biron are from TN Clinical Research Center.

## Supporting information


**Supplemental Table 1** Inclusion and Exclusion Criteria

**Supplemental Table 2** Schedule of Visits and Procedures

**Supplemental Table 3** Clinical Global Aesthetic Improvement Scale

## Data Availability

Summary data that support the findings of this study are available from the corresponding author upon reasonable request.
