# Supplementary material for: Recombinant Pure PDGF Improves Aesthetic Results and Patient Satisfaction Following RF Microneedling: A Prospective, Randomized, Controlled Clinical Trial
Source: J Cosmet Dermatol. 2025 Sep 12;24(9):e70425. doi: 10.1111/jocd.70425 (PMC12427151; doi:10.1111/jocd.70425)
Supplement: Supplementary file 1 — Supplemental Table 1 Inclusion and Exclusion Criteria [file JOCD-24-e70425-s002.pdf]

Table 1: Inclusion and Exclusion Criteria

Inclusion Criteria	Exclusion Criteria
• Mild to Moderate photodamage, Glogau Grade II or III	• Implanted devices such as pacemakers, history of arrhythmias, or known severe heart disease
• Fitzpatrick Skin Type I-VI	• Implanted metal devices in the treatment area
• Interest in receiving RF microneedling treatment	• Subjects on any medication that would affect the characteristics of the skin (medical or hormonal) within the past 3 months (e.g., Accutane)
• Current non-smoker or cessation greater than 3 years	• Any invasive or non-invasive skin treatments, hair removal, fillers or toxins performed in the past 3 months in the treated area
• Willingness and ability to provide informed consent	• Inflammatory skin conditions including but not limited to: open lacerations, abrasions, dermatitis, active acne, and psoriasis in the treatment area
• Ability to understand and comply with the requirements of the study	• History of hypertrophic scarring, keloids or abnormal wound healing, or extremely sensitive skin in the treatment area
• Negative urine pregnancy test results at the time of study entry for subjects of childbearing potential	• Pregnant, breast feeding, planning to become pregnant, or unwilling to use an accepted form of birth control during the study
	• Permanent makeup, tattoos, body piercing, or excessive hair in the treatment area
	• History of squamous cell carcinoma or melanoma in the treatment area and/or within 5 years
	• Impaired immune system due to immunosuppressive diseases such as AIDS and HIV, or current use of immunosuppressive medications
	• History of diseases stimulated by heat, such as recurrent Herpes Simplex in the treatment area
	• Excessively tanned skin, prolonged sun exposure or use of tanning beds within 2 weeks of baseline visit and throughout the study
	• Anticoagulant medications (except low dose aspirin used for prophylaxis)
	• History of allergy or hypersensitivity to any of the test product or anesthetic ingredients
	• Use of an investigational drug or device within 30 days of enrollment or participation in a research study concurrent with this study
	• As per the Investigator’s discretion, any other mental or physical condition that might make it unsafe for the subject to participate in this study
	• Currently participating or participated within 30 days prior to the start of this study in a drug or other investigational research study
