# Supplementary material for: Recombinant Pure PDGF Improves Aesthetic Results and Patient Satisfaction Following RF Microneedling: A Prospective, Randomized, Controlled Clinical Trial
Source: J Cosmet Dermatol. 2025 Sep 12;24(9):e70425. doi: 10.1111/jocd.70425 (PMC12427151; doi:10.1111/jocd.70425)
Supplement: Supplementary file 2 — Supplemental Table 2 Schedule of Visits and Procedures [file JOCD-24-e70425-s003.pdf]

Supplemental Table 2: Schedule of Visits and Procedures

Procedure	V1* Screen Day (-)14 to 0	V2* Baseline Day 0	V3 Follow-up Day 1 (+ 1 day)	V4 Follow-up Day 3 (+/- 1 day)	V5 Follow-up Day 7 (+/- 1 day)	V6 Follow-up Day 30 (+/- 1 week)
Informed Consent/Photo Release	X					
Medical History	X					
Demographics	X					
Glogau Scale Grading	X					
UPT	X					
Photos		pre/post	X	X	X	X
Treatment		X				
Pain Assessment (VAS)		X				
Subject Skin Quality Questionnaire		X				X
Subjective Tolerability Assessments		pre/post	X	X	X	X
Investigator Tolerability Assessments		pre/post	X	X	X	X
Investigator Objective Assessments		pre/post	X	X	X	X
Dispense Diary		X				
Collect Diary			X			
Subject Satisfaction Questionnaire		X				X
GAIS					X	X
Adverse Event Query		X	X	X	X	X
