# Supplementary material for: Recombinant Pure PDGF Improves Aesthetic Results and Patient Satisfaction Following RF Microneedling: A Prospective, Randomized, Controlled Clinical Trial
Source: J Cosmet Dermatol. 2025 Sep 12;24(9):e70425. doi: 10.1111/jocd.70425 (PMC12427151; doi:10.1111/jocd.70425)
Supplement: Supplementary file 3 — Supplemental Table 3 Clinical Global Aesthetic Improvement Scale [file JOCD-24-e70425-s001.pdf]

Supplemental Table 3: Clinical Global Aesthetic Improvement Scale

Rating	Description
1	**Very Much Improved** – Optimal cosmetic result in this subject
2	**Much Improved** – Marked improvement in appearance from the initial condition, but not completely optimal for this subject
3	**Improved** – Obvious improvement in appearance from initial condition, but a re-treatment is indicated
4	**No change** – The appearance is essentially the same as the original condition
5	**Worse** – The appearance is worse than the original condition
